# Overdose admissions to a district general hospital intensive care unit

**DOI:** 10.1192/bjo.2021.285

**Published:** 2021-06-18

**Authors:** Aoife Nechowska, Theophilus Samuels, Sameer Ranjan

**Affiliations:** Surrey and Sussex Healthcare NHS Trust

## Abstract

**Aims:**

This audit aimed to analyse the patient population coming into East Surrey Hospital's Intensive Care Unit from 1993 to 2019.

**Background:**

The Office for National Statistics (ONS) published a report in August 2019 on ages most likely to die by suicide and drug poisoning. Their data showed that Generation X were dying by this method in greater numbers than other age groups. This is in contrast with data from 1990s for England and Wales which showed people in their 20s were most likely to die by suicide or poisoning. This audit set out to look at admission data from an intensive care unit (ICU) in a district general hospital in Surrey over a similar period of time.

**Method:**

Patient records from 1993 to September 2019 were accessed using the WardWatcher database. To access the maxim number of admissions qualifying under the aims, the database was accessed by searching under “admission comments” for: overdose, self-harm, poison, suicide. These reports were downloaded and the lists were checked against each other to delete duplicates. This gave a total of 331 patients. The data were analysed by year, according to age, gender, season, psychiatric diagnoses and previous overdose attempts. Their outcomes were checked against recorded deaths. There was not enough information to investigate method of overdose.

**Result:**

A total of 331 patient records were accessed. The youngest patient was 15 years old, the oldest was 84 years old. The age dataset was non-parametrically distributed with the median age of 43 years (IQR 33-51 years). The age distributions for each year appeared symmetrical but total numbers for each year were small. The population was split as 191 female (58%) and 141 male (42%). 16 patients died on the ICU on admission with an overdose, 5% of total numbers, of which 19% had a previous overdose attempts and 44% had a psychiatric diagnosis. The youngest death was 22 and the oldest was 81 years old. The average age was 47 years, with the spread consistent in the 2000s and 2010s.

**Conclusion:**

The results from East Surrey Hospital's ICU do not reflect the analysis from the ONS. The mean age for each year has remained similar. Numbers for the audit were small and admission criteria to the ICU prescribe that the patient be critically unwell and may not be indicative of the total admissions to a district general hospital.

